# Fibronectin and androgen receptor expression data in prostate cancer obtained from a RNA-sequencing bioinformatics analysis

**DOI:** 10.1016/j.dib.2017.01.016

**Published:** 2017-02-03

**Authors:** Dibash K. Das, Thahmina Ali, Konstantinos Krampis, Olorunseun O. Ogunwobi

**Affiliations:** a Department of Biological Sciences, Hunter College of The City University of New York, New York, NY 10065, USA; b The Graduate Center Departments of Biology and Biochemistry, The City University of New York, New York, NY 10016, USA; c Department of Medicine, Weill Cornell Medicine, Cornell University, New York, NY 10065, USA; d Department of Physiology and Biophysics, Institute for Computational Biomedicine, Weill Cornell Medicine, Cornell University, New York, NY 10065, USA

**Keywords:** Fibronectin, Androgen receptor

## Abstract

Prostate cancer is the second most commonly diagnosed male cancer in the world. The molecular mechanisms underlying its development and progression are still unclear. Here we show analysis of a prostate cancer RNA-sequencing dataset that was originally generated by Ren et al. [3] from the prostate tumor and adjacent normal tissues of 14 patients. The data presented here was analyzed using our RNA-sequencing bioinformatics analysis pipeline implemented on the bioinformatics web platform, Galaxy. The relative expression of fibronectin (FN1) and the androgen receptor (AR) were calculated in fragments per kilobase of transcript per million mapped reads, and represented in FPKM unit. A subanalysis is also shown for data from three patients, that includes the relative expression of FN1 and AR and their fold change. For interpretation and discussion, please refer to the article, “miR-1207-3p regulates the androgen receptor in prostate cancer via FNDC1/fibronectin” [1] by Das et al.

**Specifications Table**

**Table:** 

Subject area	*Biology*
More specific subject area	*Bioinformatics*
Type of data	*Paired-end RNA-sequencing data (fastq format)*
How data was acquired	*Array Express database of the EMBL European Bioinformatics Institute*
Data format	*Pre-processed RNA-sequencing data*
Experimental factors	*The primary study performed by Ren et al. applied preprocessing steps to the RNA-sequencing reads, which were, removal of sequencing adaptors and low-quality reads with specific data preprocessing filters.*
Experimental features	*Prostate tumor and adjacent normal tissues from 14 patients were sampled for RNA sequencing and sequencing libraries were constructed using the Illumina kit following the manufacturer׳s standard protocol, which was diluted to 2.5 pM for sequencing on a single lane of an Illumina HiSeq2000 flowcell.*
Data source location	*Prostate Cancer Discovery cohort at Shanghai Changhai Hospital, Shanghai, China*
Data accessibility	*Data available in the article and at:*https://www.ebi.ac.uk/arrayexpress/experiments/E-MTAB-567/

**Value of the data**

• Performing a Bioinformatics RNA-sequencing analysis on published transcriptome data allows exploration and opportunities for discovery of new or not previously known biological implications.
• Allowing reproducibility of the analysis by using the method of the automated RNA sequencing pipeline on the Galaxy platform to analyze the same data and reusability of the pipeline to analyze other cancer transcriptome data.
• Promoting transparency of the analysis by allowing the data and methods used in the analysis accessible on databases and platforms.

## 1 Data

(Fig. 1).

## 2 Experimental design, materials and methods

We designed and implemented a comprehensive, standardized, and scalable RNA-sequencing bioinformatics analysis pipeline as a workflow on the Galaxy platform [2] (http://galaxy.hunter.cuny.edu:8080/u/bioitcore/w/ted-transcriptome-data-analysis) to analyze prostate cancer RNA-sequencing datasets from the Array Express archive of the European Bioinformatics Institute (EBI) (http://www.ebi.ac.uk/arrayexpress/ experiments/E-MTAB-567/). As described in the primary study by Ren et al., the samples comprised poly-A containing RNA sequencing paired-end reads and replicates from fourteen prostate cancer patients [3]. The poly(A) random primed containing RNA were sequenced using Illumina HiSeq 2000 at a read length of 200-250nt producing on average 400 million reads for each library. The workflow requires eight input read files, one file of the human reference genome (UCSC hg19), as well as one file of the gene annotations of the reference genome. The workflow in total performs forty-four steps, using thirteen bioinformatics tools and requires approximately 84 h on a 4 core processor server, with four stages:

1) Data Groom and Alignment. The first stage involves two steps: i) standardizing the format of the RNAseq reads using the FASTQ groomer tool and ii) read alignment against a pre-indexed human reference genome (UCSC hg19) using the Tophat2 [4] tool. The FASTQ groomer tool converts the specific sequencing formats of the FASTQ RNAseq reads to a standardized sequencing format. The Tophat2 tool uses the ultra-fast short read mapper Bowtie2, which maps reads entirely in the exons while Tophat2 identifies non-continuous mapped reads to search for junction signals in order to produce a built set of possible introns in the transcriptome. The output is a Binary Alignment Map (BAM) file of exonic reads, and also included to view the file in text format is the BAM-SAM [5] converter.
2) Differential Gene Expression Analysis. The second stage involves using the Cufflinks2 [6] software suite to reconstruct the full set of transcripts and quantify their amounts. Cufflinks uses the BAM alignment file from TopHat2 and a reference gene annotation file in Gene Table Format (GTF) to generate a transcriptome assembly. The assemblies from the cancer and normal samples are merged together using the Cuffmerge utility supplied with the same reference gene annotation file. The Cuffcompare tool was used to validate the assembled transcripts produced from Cufflinks and compare the assembled transcripts from the merged assembly to the reference gene annotation to report new assembled transcripts of the reads and gene isoforms, creating as output a new annotation GTF file. The Cuffcompare GTF file is then processed by Cuffdiff2 [6] along with the alignment BAM files that serves as the replicates, in order to calculate expression levels in fragments per kilobase of exon per million fragments mapped (FPKM). Cuffdiff also tests the statistical significance of the observed changes and accounts for the variability in measurements of an experiment. It reports results of differential expression analysis in eleven text files.
3) Variant Analysis: The third stage involves three steps: i) identifying single genetic variants for each position of the read bases against the reference genome using the SAMtools mpileup [5], [7] tool ii) calling the somatic and germ line mutations of the single genetic variants using the VarScan [6], [7], [8] tool and iii) annotate and predict the effects of the single genetic variants using the SnpEff package. The SAMtools mpileup tool uses the alignment BAM files with a reference genome, and generates a “pileup” of read bases, in which the VarScan tool uses its variant calling algorithm to call mutations on the mpileup files and reports results in a Variant Call Format (VCF) tabular file. The SnpEff program uses its collection of variant annotation and effect prediction tools to annotate the called genetic variants in the VCF files based on their genomic locations and categorizes and predicts their coding effects.
4) Isoform-Level Analysis: The fourth stage involves two steps i) detecting chimeric transcripts, RNAs encoded by a fusion gene or by two different genes using the ChimeraScan tool and ii) quantifying alternative splicing events using the SpliceTrap tool. The ChimeraScan tool aligns paired-end reads using Bowtie2, to a combined genome-transcriptome reference and potential fusion breakpoints arise from fragments that align to distinct references or distance genomic locations in the same reference [9]. The main output file is a tabular text file named chimeras.bedpe. The SpliceTrap tool measures exon inclusion ratios in paired-end RNA-seq data and outputs a text file of its gene splicing locations and quantity [10]. Please refer to the article, “miR-1207-3p regulates the androgen receptor in prostate cancer via FNDC1/fibronectin” [1] by Das et al. for analysis and discussion [1].

## Figures and Tables

**Fig. 1:**
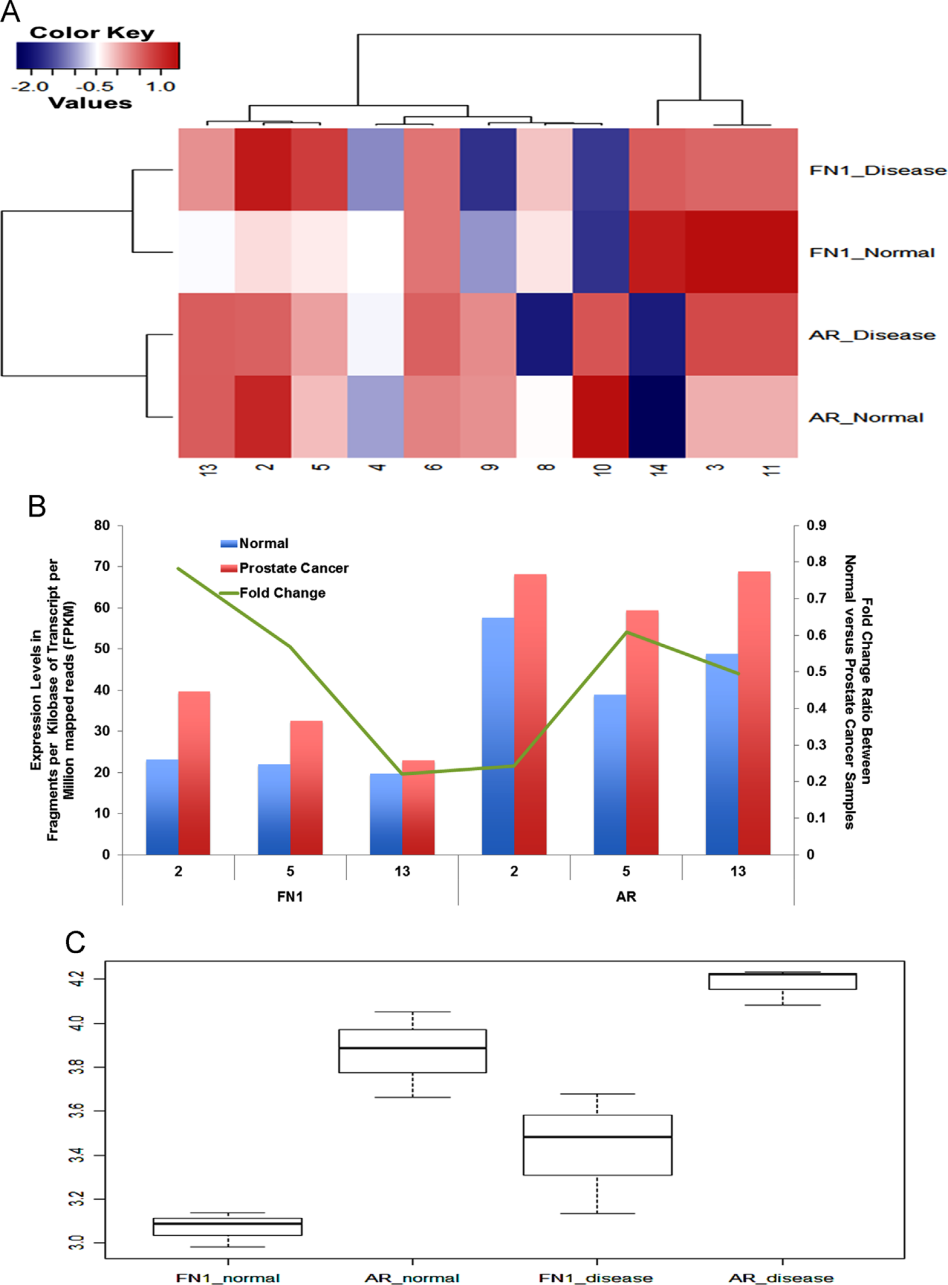
(A) Heatmap representing the strength of association between FN1 and AR in the samples from 11 patients with reliably analyzable gene expression patterns (*P*<0.05). Similar color patterns represent a strong correlation. Upregulation of genes is denoted by increasing color from blue to red. (B) 3 out of 14 patients from a Chinese study showed positive correlation between FN1 and AR (patients 2, 5 and 13). (C) Boxplot of relative gene expression of FN1 and AR in normal versus prostate cancer samples (patients 2, 5 and 13).
